# Predicting the UV–vis spectra of oxazine dyes

**DOI:** 10.3762/bjoc.7.56

**Published:** 2011-04-15

**Authors:** Scott Fleming, Andrew Mills, Tell Tuttle

**Affiliations:** 1 WestCHEM, Department of Pure and Applied Chemistry, University of Strathclyde, 295 Cathedral Street, Glasgow G1 1XL, UK

**Keywords:** DFT, dyes, oxazine, TD-DFT, UV–vis

## Abstract

In the current work we have investigated the ability of time-dependent density functional theory (TD-DFT) to predict the absorption spectra of a series of oxazine dyes and the effect of solvent on the accuracy of these predictions. Based on the results of this study, it is clear that for the series of oxazine dyes an accurate prediction of the excitation energy requires the inclusion of solvent. Implicit solvent included via a polarizable continuum approach was found to be sufficient in reproducing the excitation energies accurately in the majority of cases. Moreover, we found that the SMD *s*olvent *m*odel, which is dependent on the full electron *d*ensity of the solute without partitioning into partial charges, gave more reliable results for our systems relative to the conductor-like polarizable continuum model (CPCM), as implemented in Gaussian 09. In all cases the inclusion of solvent reduces the error in the predicted excitation energy to <0.3 eV and in the majority of cases to <0.1 eV.

## Introduction

**Figure 1:**
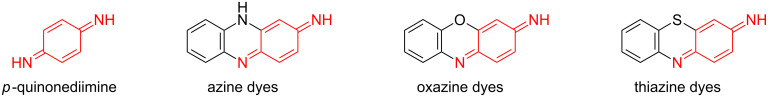
Quinone imine structural relationships.

Oxazine dyes are a subclass of quinone imines, which are all based upon the *p*-benzoquinone imine or -diimine scaffold. Other important subclasses within the quinone imines include, the azine dyes and thiazine dyes. The structural relationships described are illustrated in Figure 1 for clarity.

All the dyes are based on an anthracene skeleton in which one carbon is replaced by a nitrogen atom and another by an additional heteroatom such as N, O, or S in the central ring. Although azine dyes have been found to demonstrate solvatochromism [1–3], and many thiazine dyes are metachromatic [4–5], this investigation is limited to the study of oxazine dyes.

**Figure 2:**
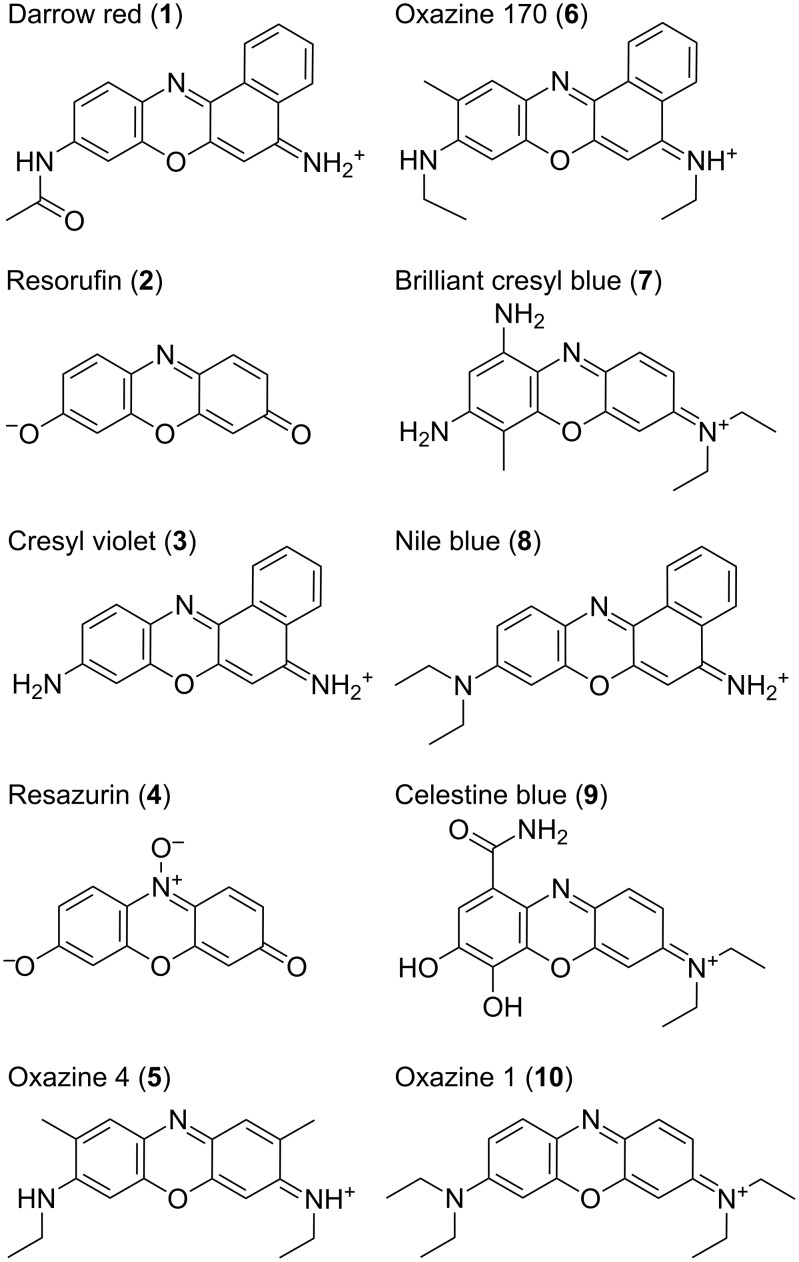
Numbering and structure of oxazine dyes studied in this work (counterions not shown).

Oxazine dyes are useful partly because they exhibit solvatochromism, this makes them sensitive to their surrounding environment, and hence they have been utilized in various applications as molecular probes [6–8]. In the current investigation we focus on the ten oxazine dyes shown in Figure 2, which are readily soluble in aqueous solution, in order to determine the ability of computational methodology to describe the solvent dependence on the absorption maxima.

Solvatochromism in oxazine dyes has been observed and the mechanism(s) explored in previous studies by various authors [9–11]. Most of these investigations have involved measuring experimentally the spectroscopic features of oxazine dyes, upon varying the solvent polarity. However, some attempt has been made to rationalize these observations by a computational study of the solvatochromism of the oxazine dye Nile red [12]. In the investigation, TD-DFT was applied in order to try and explore the contributing factors in the solvatochromism observed with Nile red, upon gradually increasing the solvent polarity from benzene to acetonitrile. A computational approach such as this is advantageous, because it allows consideration of the individual molecular orbital transitions involved – a feat difficult to achieve via experiment alone.

TD-DFT has become the modern day workhorse for the determination of electronic excited states in medium sized systems (up to 100 atoms) [13–17]. This method performs particularly well for predicting the excitation energies of local excitations and Rydberg states (although in the case of Rydberg states the choice of functional is particularly important with accurate excitation energies for these states requiring large amounts of exact exchange), while its performance in predicting charge-transfer (CT) states is more questionable [18–23]. A number of studies have demonstrated the failures of TD-DFT in predicting CT excitation energies accurately [24–29], while one can also find within the literature examples where TD-DFT performs well for such states [30]. As such in the current work we explore the ability of various density functionals to predict the excitation energies of the intramolecular CT states in a series of oxazine dyes and the effect of solvent models on the accuracy of these predictions. The assessment of these functionals was carried out against the experimental λ_max_ for the absorption of each dye, which was determined experimentally.

## Results and Discussion

### Role of the solvent

The gas-phase optimized geometries of the dyes were used as the basis for the single point excited state calculations. The six lowest singlet vertical excitation energies and oscillator strengths from the TD-DFT calculations were used to predict the UV–vis spectrum for each dye through the fitting of a Gaussian (with the GaussView default parameters for half-width) centered at the computed excitation energies. The predicted UV–vis spectra in the gas-phase, gas//CPCM, and gas//SMD were plotted for each dye, and the λ_max_ values in each case are summarized in Table 1.

**Table 1:** Calculated λ_max_ values and the shift resulting from the different solvent models.^a^

Dye	λ_max_			Shift	
	Gas^b^	CPCM^c^	SMD^d^	CPCM^c^	SMD^d^

**1**	485	548	566	62	81
**2**	458	546	571	88	113
**3**	481	563	587	82	106
**4**	486	570	596	84	110
**5**	468	558	588	90	120
**6**	505	597	626	92	121
**7**	470	596	616	129	146
**8**	512	597	625	85	113
**9**	476	560	584	86	108
**10**	492	584	617	92	125

^a^λ_max_ and the shift are reported in nm. The shifts are reported relative to the gas-phase λ_max_. ^b^Gas refers to the gas-phase. ^c^CPCM refers to the TD-DFT single point calculations using the CPCM solvent model on the gas-phase optimized structures. ^d^SMD refers to the TD-DFT single point calculations using the SMD solvent model on the gas-phase optimized structures.

The results in Table 1 indicate a red shift of about 60–130 nm upon moving from the gas-phase to the CPCM solvent description. Upon moving from the CPCM to the SMD description of the solvent we observe a further red shift in the λ_max_ value relative to the gas-phase calculated spectra. This result is consistent with a narrowing of the energy gap between ground and excited states, due to an increased stabilization of the more polarizable excited state by polar solvents. Clearly, the SMD description of the solvent provides greater stabilization of the excited state with the red shift increased on average by 20–30 nm relative to the spectra obtained within the CPCM solvent model.

The absorbance spectra for oxazine dyes are often described as occurring through CT excitations. Therefore, it is also helpful to examine the dipole moments, and corresponding transition dipole moments in the gas-phase and in the solvent phase. In each case, the transition moments chosen relates to the most significant excited state (vida infra). In the first instance an examination of the dipole and transition moment magnitudes, shows a increase upon moving to the solvated species (Table 2).

**Table 2:** Magnitudes of dipole and transition moments in Debye.^a^

Dye	Dipole		Transition	
	Gas	CPCM	Gas	CPCM

**1**	8.35	12.44	3.41	4.75
**2**	3.72	5.21	3.28	4.66
**3**	3.99	5.81	3.17	4.65
**4**	3.16	4.40	3.07	4.34
**5**	2.27	3.10	3.69	4.91
**6**	3.13	4.65	3.74	5.06
**7**	2.74	3.79	3.11	3.55
**8**	3.24	4.94	3.59	4.98
**9**	11.82	16.61	3.21	4.51
**10**	2.16	2.99	4.00	5.19

^a^Gas refers to the gas-phase. B3LYP TD-DFT calculations and CPCM refers to the solvent phase CPCM/B3LYP TD-DFT calculations. All TD-DFT calculations were carried out on the gas-phase B3LYP/6-311++G(d,p) optimized geometries.

**Figure 3:**
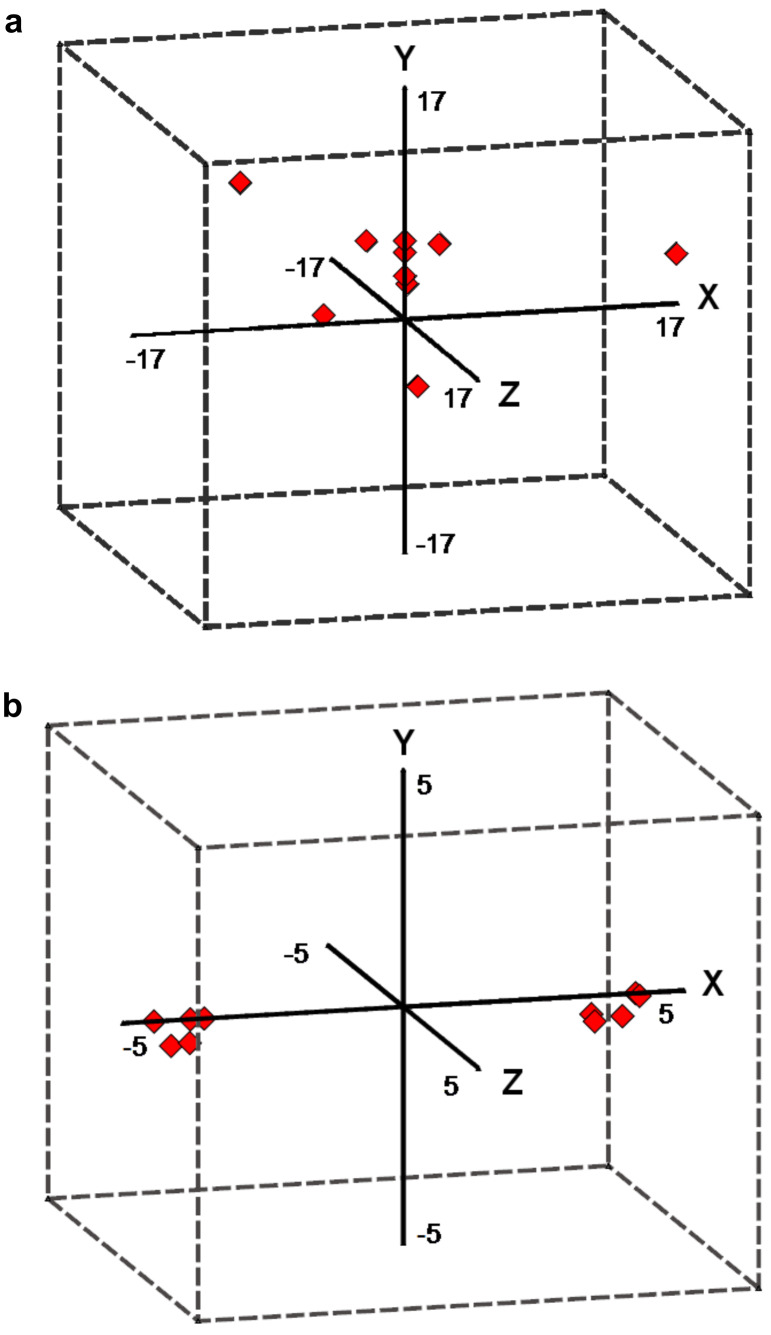
Directions of solvated (a) dipole moments and (b) transition moments from origin (0,0,0).

The observed increases in magnitude are indicative of polarization by the solvent field. It is also possible to examine the x, y, and z components associated with the corresponding dipole and transition moments (Figure 3a and Figure 3b). It should be noted, that the direction associated with each molecule does not change significantly between gaseous and solvated phases, and hence plots are only shown for the latter.

In the above representation the molecule lies in the xy plane, and is elongated along the x-axis, hence the z component only makes a very slight contribution towards the overall direction. In the case of the ground state dipole moments (Figure 3a), the observed vector differs depending upon the substituents present. For instance, Oxazine 1 (**10**), Oxazine 4 (**5**), Resazurin (**4**), and Resorufin (**2**) have symmetry in the yz plane, and thus have vectors based almost exclusively on the y-axis. In more complex examples, the direction of dipole moment vector is predominately dictated by the positions of the amines/imines, which possess a partial positive charge due to electron donation to the aromatic system. In contrast the transition dipole moments (Figure 3b) show very little variation in the magnitude and direction associated with the transition moment. The only significant contribution lies along the x-axis, and in each case the magnitude is consistently 3–4 Debye. This is indicative of CT along the extended aromatic system and consistent with the classical description of these excitations.

### Performance of functionals and solvent models

The TD-DFT calculations were carried out using the selection of functionals and solvation methods described in the computational methods. The accuracy of the calculated λ_max_ values was assessed against the values obtained experimentally (Table 3).

**Table 3:** Comparison between the experimental and calculated λ_max_ values at different levels of theory.^a^

Dye	Exp.	B3LYP^b^	B3LYP^c^	B3LYP^d^	CAM-B3LYP^d^	M06^d^	M06-L^d^	M06-2X^d^

**1**	502	486	548	566	535	565	571	532
**2**	572	458	546	571	581	578	554	578
**3**	588	481	563	587	573	591	582	570
**4**	602	486	570	596	591	601	578	583
**5**	616	468	558	588	580	592	579	576
**6**	620	505	597	626	610	629	622	607
**7**	624	467	596	616	592	613	625	590
**8**	636	512	597	625	608	625	621	607
**9**	646	474	560	584	560	586	606	557
**10**	654	492	584	617	606	616	606	606
MSE		−123	−34	−8	−22	−6	−12	−25
MUE		123	43	22	31	23	26	33

^a^All wavelengths are given in nm. MSE is the mean signed error and MUE is the mean unsigned error relative to the experimental λ_max_. ^b^Gas-phase TD-B3LYP/6-311++G(d,p). ^c^CPCM/TD-B3LYP/6-311++G(d,p). ^d^SMD/TD-DFT/6-311++G(d,p). All single point TD-DFT calculations employed the gas-phase optimized structures.

The data presented in Table 3 clearly shows the important role of the solvent in attaining a realistic description of the excited state. The gas-phase calculated λ_max_ values are all strongly blue shifted relative to the experimental data with an average error of 123 nm (0.51 eV). The inclusion of the solvent using either of the continuum models (i.e., either the CPCM or SMD model) corrects this error to a large degree with the mean unsigned error (MUE) decreased to 43 nm (0.15 eV) with the CPCM solvent model and 22 nm (0.08 eV) within the SMD solvent model at the B3LYP level of theory. Given the large transition dipole moments for the transitions corresponding to the λ_max_ excitations (Table 2), we examined whether a number of functionals that have been shown to perform well for CT states could improve upon the TD-B3LYP calculated excitation energies.

Within the SMD solvent model, TD-B3LYP performs well across all of the dyes. However, the largest errors in the calculated λ_max_ values are found for dyes **1** (Darrow red), which is overestimated by 64 nm (−0.28 eV); and **9** (Celestine blue), which is underestimated by 62 nm (0.20 eV). In the case of **1** the best performing functional is M06-2X, which still overestimates the value of λ_max_ (30 nm; −0.14 eV) but to a lesser extent relative to B3LYP. However, across the series of dyes, M06-2X is the worst performing functional with an MUE of 33 nm (0.11 eV) and has the largest error for **9** (89 nm; 0.31 eV). In contrast, the M06-L functional provides the most accurate representation of **9**, underestimating the λ_max_ excitation by 40 nm (0.13 eV), however, offers no improvement in the prediction of the λ_max_ value of **1** (M06-L error: 69 nm; −0.30 eV). Surprisingly, the gas-phase calculated value of λ_max_ for **1** is relatively accurate. The gas-phase B3LYP calculation of **1** underestimates the value of λ_max_ by only 16 nm (0.08 eV), although this appears to be a fortuitous error cancellation as the solvent calculations systematically result in a strong red shift, which leads to the overestimation mentioned above.

**Figure 4:**
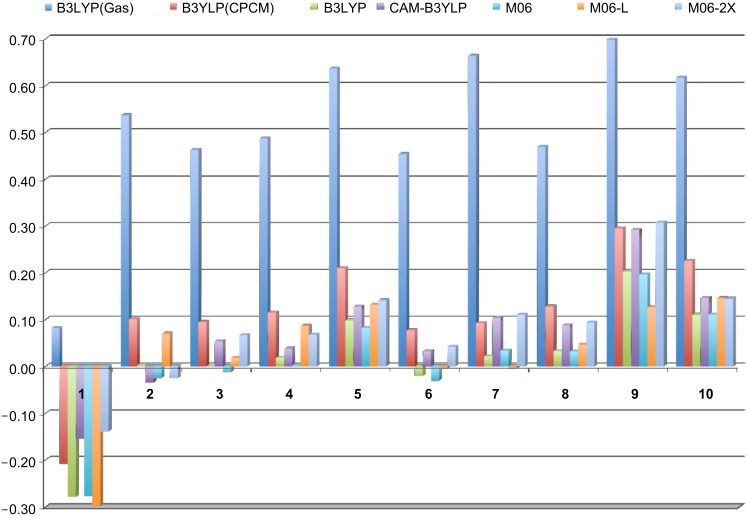
Error between experimental and calculated λ_max_ values for each dye at the different levels of theory investigated. All errors are reported in eV. All structures were optimized in the gas-phase at the B3LYP/6-311++G(d,p) level of theory. B3LYP(Gas) refers to the TD-B3LYP calculation in the gas-phase. B3LYP(CPCM) refers to the TD-B3LYP calculation within the CPCM solvent model. All other TD-DFT calculations were carried out using the SMD solvent model as described in the computational methods.

Despite the difficulties associated with predicting the excitation energies of **1** and **9**, the range of different functionals that were tested perform remarkably well once the effect of solvent is taken into account. This is clearly seen in the plot of the errors for the various methodological combinations (Figure 4). Given the non-linear relationship between the observed wavelength and the excitation energy (i.e., an error at a high excitation energy will have a lesser impact on the calculated λ_max_ than an equally sized error at a lower excitation energy), the errors associated with the computed excitation energies at the various levels of theory, shown in Figure 4, are in eV. Increasing the percentage of HF exchange is considered beneficial for low-lying excited states that have an ionic character and as such suffer, to a greater extent, from a self-interaction error. This error can be corrected to some extent by increasing the percentage of HF exchange [19]. However, in our dyes, this was not observed, as both the CAM-B3LYP (greater HF exchange at long range) and M06-2X functionals produce larger errors (Figure 4). Clearly in the series of dyes examined, the increased HF exchange leads to a slight over-correction, which has also been observed by others [22].

The calculated transition dipole moments for the various dyes are consistent with a CT nature of the excitation. Moreover, the largest errors are obtained for **1** and **9**, which also have significantly larger ground state dipole moments, relative to the other dyes. TD-DFT is well-known to fail in a variety of CT excitations, which is in contrast to the results obtained for the other eight dyes. Therefore, we employed the Tozer diagnostic to examine whether the calculated excitation energies indeed correspond to CT excitations from an orbital overlap perspective.

### Orbital overlap

Within the literature there are conflicting cases as to the accuracy of TD-DFT in predicting CT states [24–30]. In some cases, TD-DFT methods appear to perform reasonably well, whereas in other cases – generally long-range CT – TD-DFT significantly underestimates the excitation energy. In an effort to help identify those excitations where TD-DFT is likely to fail, Tozer and co-workers have recently introduced the use of an orbital overlap diagnostic which utilizes the spatial overlap of the unperturbed ground state orbitals in order to assess the likelihood of an accurate TD-DFT excitation energy, for local, Rydberg and intramolecular CT excitations between those orbitals.

The diagnostic, Λ, considers the spatial overlap between the orbitals involved in the excitation. Where more than one set of orbitals contribute to the excitation (as commonly occurs in TD-DFT calculations) the orbital overlaps are scaled by the contribution (κ) from each pair. In the following, we have employed the same form of Λ as that introduced by Tozer to investigate the spatial overlap between our orbital pair, namely:

[1]
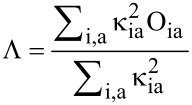


where the spatial overlap (O_ia_) between the occupied orbital (φ_i_) and the virtual orbital (φ_a_) is given by the inner product of the moduli of the two orbitals:

[2]
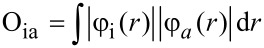


and κ_ia_ is the largest coefficient in the CI expansion for each orbital pair.

**Figure 5:**
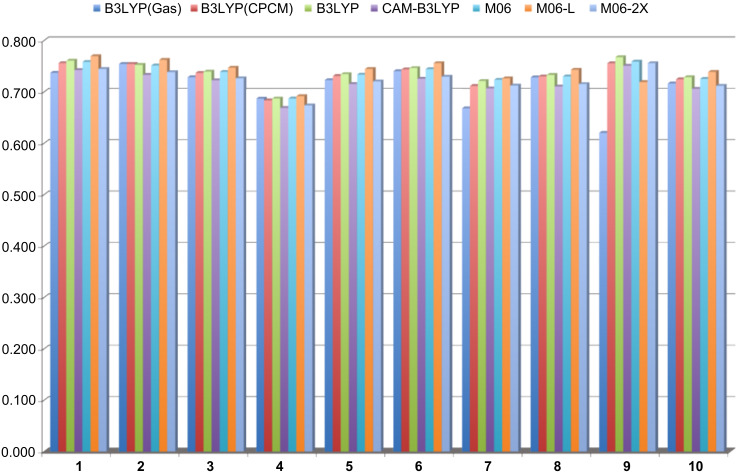
The orbital overlaps (Λ) for each dye at the different levels of theory investigated. All structures were optimized in the gas-phase at the B3LYP/6-311++G(d,p) level of theory. B3LYP(Gas) refers to the TD-B3LYP calculation in the gas-phase. B3LYP(CPCM) refers to the TD-B3LYP calculation within the CPCM solvent model. All other TD-DFT calculations were carried out using the SMD solvent model as described in the computational methods.

The resulting overlaps calculated at the various level of theory for each dye are plotted in Figure 5. The value of Λ is largely conserved across the different methods for each dye. In the case of **9**, where there is some variation between the values of Λ calculated in either the gas or solvent phase, this is due primarily to the difference in the two orbital pairs that contribute to the λ_max_ excitation. For the primary excitation in **9**, the κ_ia_ value for the minor contributing orbital pair (HOMO-3–LUMO in the gas-phase and HOMO-1–LUMO in the solvent phase) varies between 0.4–0.6, while the major contribution (HOMO–LUMO) remains constant across the series, resulting in the slight variation in the Λ values observed for this dye. Figure 5 illustrates that the orbital overlap for the solvent phase calculations is largely unaffected by the choice of functional. This is due primarily to the fact that the predominant contribution to the excitation energy and the nature and overlap of the orbital pair (HOMO–LUMO) is conserved across the different methods. In the gas-phase TD-DFT calculations the number of orbital pairs contributing towards the λ_max_ excitation varies with respect to the solvent calculations – two orbital pairs contribute in the gas-phase calculations for **2**, **3**, **5**, **7**, **9**, and **10** – however, a comparable Λ value is obtained in most cases due to the dominance of the HOMO–LUMO contribution in these excitations as well.

The strong overlap between the occupied and virtual orbital can intuitively be seen by visualizing the orbitals. Given the consistency of the calculated overlaps the orbital contributions to the λ_max_ for each dye across the series of functionals, only the orbitals calculated at the CPCM/B3LYP level of theory are displayed in Table 4.

**Table 4:** Orbital pairs involved in the λ_max_ excitation for each dye.^a^

Dye	Occupied MO(s)	Virtual MO(s)

**1**	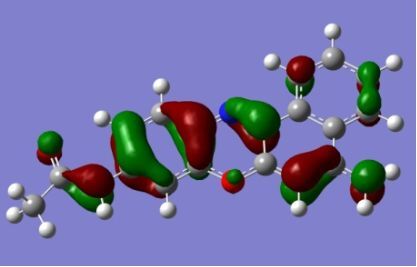	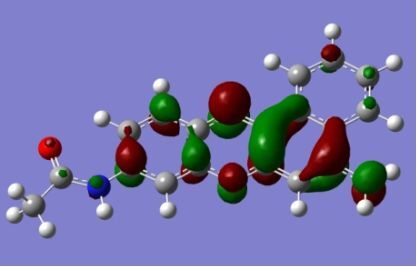
**2**	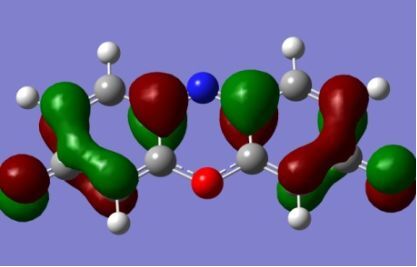	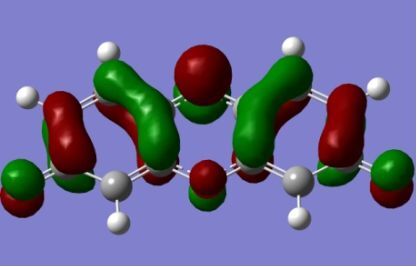
**3**	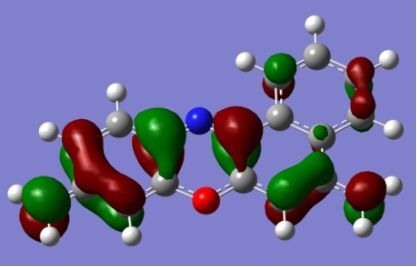	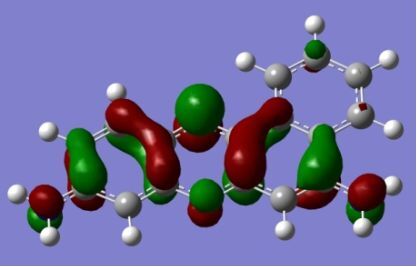
**4**	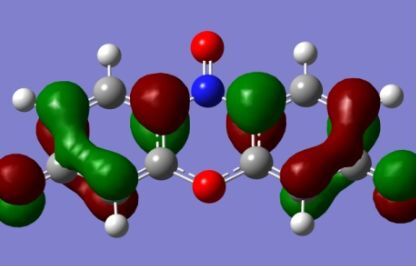	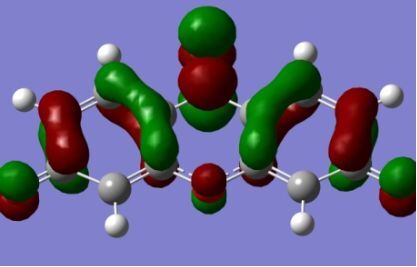
**5**	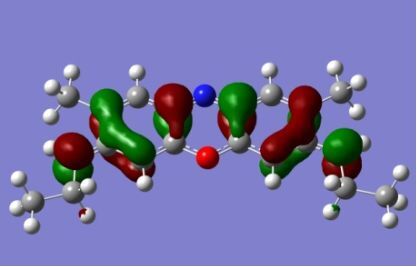	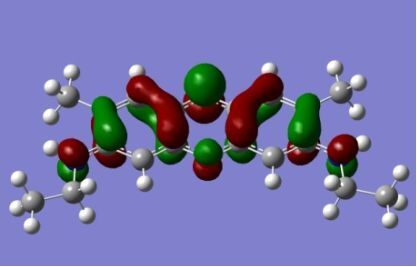
**6**	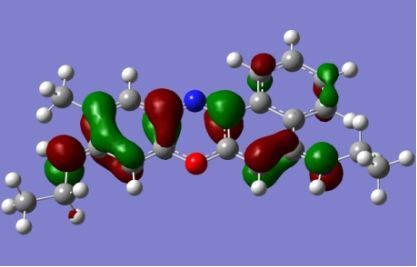	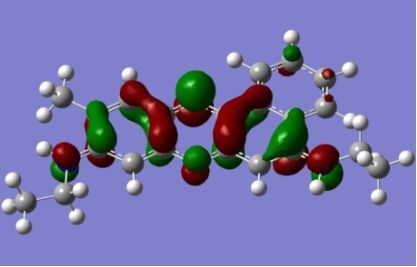
**7**	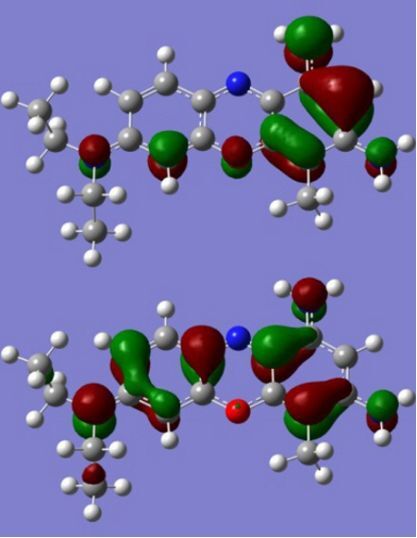	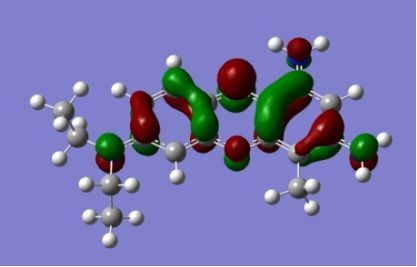
**8**	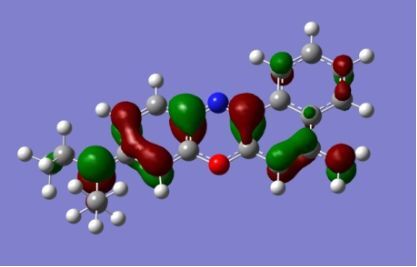	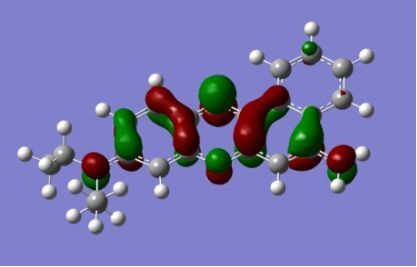
**9**	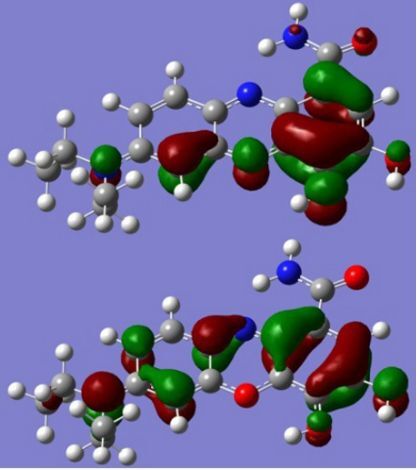	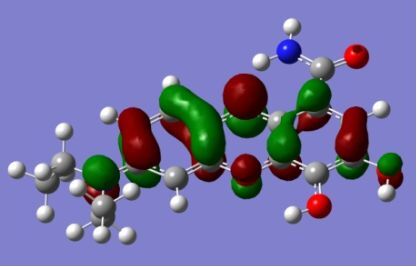
**10**	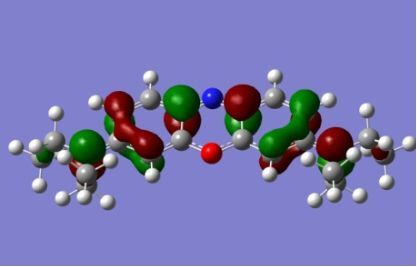	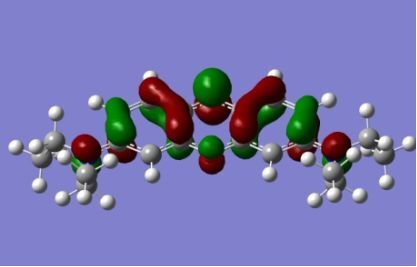

^a^All orbitals are taken from the CPCM/B3LYP/6-311++G(d,p) single point calculation. Isovalue for surface = 0.04.

Tozer and co-workers suggest that an overlap of Λ ≤0.3, indicates that TD-DFT will struggle to predict correctly the excitation energy in such cases which can be classified as problematic CT states. The calculated orbital overlaps in our series of oxazine dyes are all above the cut-off value, which is consistent with the general accuracy of the calculated excitation energies and suggests that the excitations do not fall into the category of being CT states. However, it is interesting to note that the overlap diagnostic does not cover all cases where TD-DFT fails to predict accurately the λ_max_. The most striking example of this is **1**, which has strong orbital overlap and the largest error. However, as the authors of the diagnostic point out, “the test just states that low Λ implies large errors, it does not preclude the possibility that high Λ can also have large errors” [31].

## Conclusion

In the current work we have investigated the ability of TD-DFT to predict the absorption spectra of a series of oxazine dyes and the effect of solvent on the accuracy of these predictions. Based on the results of this study, it is clear that for the series of oxazine dyes and accurate prediction of the excitation energy requires the inclusion of solvent. Implicit solvent included via a polarizable continuum approach was found to be sufficient in reproducing the excitation energies accurately in the majority of cases. Moreover, we found that the SMD solvent model gave more reliable results for our systems relative to the CPCM model, as implemented in Gaussian 09.

This study has also illustrated that for the oxazine dyes studied the principal excitation can be classified as an intramolecular CT excitation, based on the transition dipole moments of the excitations. Nonetheless, in all cases the inclusion of solvent reduces the error in the predicted excitation energy to <0.3 eV and in the majority of cases to <0.1 eV.

## Experimental

The commercially available oxazine dyes were used as supplied from Aldrich. Depending upon the solubility of the dye, deionized water was used as the solvent for all dyes. Counterions varied as summarized in Table 5. All absorption spectra were obtained using 1 cm cuvettes in a Cary 50 UV–vis spectrophotometer, scanning within the 200–800 nm range. Solutions of 10^−4^ mol dm^−3^ were prepared in a 100 cm^3^ volumetric flask, and, if required, subsequently diluted by a factor of ten so as to obtain a maximum absorbance of less than 1.

**Table 5:** Counterions of each oxazine dye.

Dye	Counterion

Nile blue	SO_4_^2−^
Brilliant cresyl blue	ZnCl_4_^2−^
Cresyl violet	MeCOO^−^
Oxazine 1	ClO_4_^−^
Oxazine 4	ClO_4_^−^
Oxazine 170	ClO_4_^−^
Celestine blue	Cl^−^
Darrow red	Cl^−^
Resazurin	Na^+^
Resorufin	Na^+^

### Computational methods

All structures were optimized in the gas-phase. For geometry optimizations, the B3LYP [32–37] level of theory with the 6-311++G(d,p) basis set [38–39] was employed and no symmetry constraints were imposed. Time dependent density functional theory [13–17] (TD-DFT) single-point calculations were performed on the optimized structures to obtain the calculated λ_max_ values. The TD-DFT calculations were carried out in both the gas-phase and the aqueous phase. In order to evaluate the effect of the description of the solvent on the calculated spectra, both the conductor-like polarizable continuum model [40–41] (CPCM) and SMD [42] (Truhlar’s new solvent model, which is dependent on the full electron density of the solute without partitioning into partial charges) solvent models were employed within the TD-DFT calculations. The ability of different density functionals to accurately describe the excited states of the oxazine dye series was investigated by varying the functional employed in the single point TD-DFT calculations using the B3LYP optimized geometry of the molecule. The series of functionals investigated in this way includes B3LYP, CAM-B3LYP [43] (the coulomb attenuated version of the B3LYP functional which has been shown to provide a better description of CT states) [28], M06 [44], M06-L [45], and M06-2X [44] as the M06 suite of functionals have been shown to perform generally well for a range of molecular properties [46]. The M06-2X functional was included to examine the effect of an increased percentage of HF exchange on the ability of the functional to predict accurately the excitation energies as this has been shown to be beneficial in some cases [19]. All calculations were done within the Gaussian 09 program [47]. Finally, we have also employed the orbital overlap diagnostic of Tozer et al*.* in order to assess the CT character in the principal excited states [27].
